# Clustering More than Two Million Biomedical Publications: Comparing the Accuracies of Nine Text-Based Similarity Approaches

**DOI:** 10.1371/journal.pone.0018029

**Published:** 2011-03-17

**Authors:** Kevin W. Boyack, David Newman, Russell J. Duhon, Richard Klavans, Michael Patek, Joseph R. Biberstine, Bob Schijvenaars, André Skupin, Nianli Ma, Katy Börner

**Affiliations:** 1 SciTech Strategies, Inc., Albuquerque, New Mexico, United States of America; 2 University of California Irvine, Irvine, California, United States of America; 3 NICTA Victorian Research Laboratory, Melbourne, Australia; 4 School of Library and Information Science, Indiana University, Bloomington, Indiana, United States of America; 5 SciTech Strategies, Inc., Berwyn, Pennsylvania, United States of America; 6 Collexis, Inc., Geldermalsen, The Netherlands; 7 Department of Geography, San Diego State University, San Diego, California, United States of America; Indiana University, United States of America

## Abstract

**Background:**

We investigate the accuracy of different similarity approaches for clustering over two million biomedical documents. Clustering large sets of text documents is important for a variety of information needs and applications such as collection management and navigation, summary and analysis. The few comparisons of clustering results from different similarity approaches have focused on small literature sets and have given conflicting results. Our study was designed to seek a robust answer to the question of which similarity approach would generate the most coherent clusters of a biomedical literature set of over two million documents.

**Methodology:**

We used a corpus of 2.15 million recent (2004-2008) records from MEDLINE, and generated nine different document-document similarity matrices from information extracted from their bibliographic records, including titles, abstracts and subject headings. The nine approaches were comprised of five different analytical techniques with two data sources. The five analytical techniques are cosine similarity using term frequency-inverse document frequency vectors (tf-idf cosine), latent semantic analysis (LSA), topic modeling, and two Poisson-based language models – BM25 and PMRA (PubMed Related Articles). The two data sources were a) MeSH subject headings, and b) words from titles and abstracts. Each similarity matrix was filtered to keep the top-n highest similarities per document and then clustered using a combination of graph layout and average-link clustering. Cluster results from the nine similarity approaches were compared using (1) within-cluster textual coherence based on the Jensen-Shannon divergence, and (2) two concentration measures based on grant-to-article linkages indexed in MEDLINE.

**Conclusions:**

PubMed's own related article approach (PMRA) generated the most coherent and most concentrated cluster solution of the nine text-based similarity approaches tested, followed closely by the BM25 approach using titles and abstracts. Approaches using only MeSH subject headings were not competitive with those based on titles and abstracts.

## Introduction

Document clustering is important for a variety of information needs and applications such as collection management, summary and analysis. For example, funding agencies continually need to analyze collections of grant proposals for research portfolio analysis. Document clustering algorithms use and require some definition of distance or similarity between pairs of documents. Different document similarity approaches have been investigated in the context of information retrieval, which defines similarity as a relevance or ranking function [1], [2], [3], [4] typically optimized to maximize precision and/or recall. Despite early efforts showing that document retrieval and document clustering are highly linked topics [5], [6], [7], most recent work using similarity measures is focused on improving the relevancy and ranking of search results [8], [9], [10] with little or no reference to the important task of clustering.

This focus on information retrieval is not surprising given the overwhelming increase in the number and variety of documents available over the Internet, and through portals to scholarly literature such as the Web of Science, Scopus, and MEDLINE. The use of search engines is far more a part of our lives than is the use of clustered document sets. This is as true in the world of biomedical literature as it is for any other literature; most studies related to enhancing the results of MEDLINE searches are very similar in nature to those being done in the broader information retrieval community [11], [12], [13]. The TREC conferences with their associated tasks and test collections have been a significant part of this effort [14]. Clustering and the accuracy of clusters remain secondary issues to that of relevance when similarity approaches are explored in the context of biomedical literature [15], [16], [17].

There are an increasing number of practical applications involving document sets where retrieval of a small set of relevant documents does not suffice but the entire dataset must be examined for inherent structures, e.g., clusters of similar documents. For example, portfolio analysis by agencies, companies, and universities requires partitioning of their portfolios (e.g. grants, publications, patents) into coherent and organizationally meaningful groups prior to the computation and reporting of metrics for each group. The same similarity approaches (known as relevance and ranking functions in the context of search and retrieval) that are being used to rank search results can also be used to cluster document sets.

Although different similarity approaches have been explored in a search context as referenced above, such comparisons in a clustering context have only started to appear in the literature. Some studies compare different textual similarity approaches to email classification for spam detection [18], [19]. Other studies using scientific articles compare citation-based approaches (e.g., co-citation analysis, bibliographic coupling), text-based approaches (e.g., tf-idf, latent semantic analysis) and hybrid measures, all on relatively small scales (one study used only 43 documents [20], [21], others just thousands of documents [22], [23], [24], [25]). Results have been mixed [26], with citation-based approaches performing best in some studies, text-based approaches in others, and hybrids in yet others. There is no particular pattern in the conflicting results, other than that the differences are likely field-specific. Given the mixed results to date, we consider the clustering accuracies of different similarity approaches to be an open and unanswered research question, especially at large scale.

Our study was thus designed to seek a robust answer to the question of which similarity approach would provide the most accurate cluster solution of a large biomedical literature set of over two million documents. We equate accuracy with the notion of cluster quality; clusters in which the contents are all very similar to each other are of higher quality than clusters where the contents are different from each other. We measure cluster quality using a textual coherence measure based on the Jensen-Shannon divergence [27], and using concentration measures based on the grant-to-article linkages indexed in MEDLINE. The full study compared three citation-based approaches, nine text-based approaches, and one text-citation hybrid approach. Due to the size and wide scope of this study, the citation and hybrid approaches are reported in another article [26]; results of the text-based approaches are reported here. Among the text-based approaches, two stood out as superior to the others: PubMed's own related article approach (PMRA) and the BM25 approach using titles and abstracts.

## Methods

In this study we used the following process:

1. define a corpus of documents,
2. extract and pre-process the relevant textual information from the corpus,
3. calculate pairwise document-document similarities using nine different similarity approaches,
4. create similarity matrices keeping only the top-n similarities per document,
5. cluster the documents based on this similarity matrix, and
6. assess each cluster solution using coherence and concentration metrics.

Each of these process steps is described in detail here.

### Study corpus

Given that our study investigated both text-based and citation-based techniques, we needed a corpus of documents that could be used to compare the two. This required both textual and citation information for each individual record. We also included MeSH terms (PubMed's medical subject headings) given the widespread use of these descriptors among the biomedical community and the NIH. No single database contains all of this information. Thus, to build a corpus with titles, abstracts, MeSH terms, and reference lists, we matched and combined data from the MEDLINE and Scopus (Elsevier) databases. The resulting set was then limited to those documents published from 2004-2008 that contained abstracts, at least five MeSH terms, and at least five references in their bibliographies, resulting in a corpus comprised of 2,153,769 unique scientific documents (Supporting Information S1).

### Text extraction and pre-processing

MeSH terms and words from titles and abstracts were extracted from a version of MEDLINE dated September 1, 2009 for all documents in the corpus. PubMed IDs (PMID) were used as the unique document identifiers.

For MeSH terms, qualifiers were ignored and all Class 3 (check tags) and Class 4 (geographical locations) terms were removed. In addition, all leading ‘*’ characters were stripped. MeSH terms were then used verbatim without any further tokenization; those that occurred in fewer than four documents were ignored. The result of this processing was a MeSH-document matrix consisting of 23,347 unique MeSH terms and 2,153,769 documents with 25,901,212 MeSH-document pairs.

Titles and abstracts (TA) were processed differently. After concatenating the title and abstract for each document, all punctuation characters except apostrophes were removed from the text and replaced with a single space. The resulting text was converted to lower case and split on whitespace, leaving only tokens with no whitespace, and no empty tokens. Each token with a standard contraction was then separated into a root and a contraction (e.g., don't – do not). Contractions were then removed since all such suffixes are forms of words found on standard stopword lists or are possessive forms of other words. Tokens appearing on our stopword list (the official MEDLINE stopword list of 132 words plus a list of 300+ words commonly used at NIH, available at http://sts.cns.iu.edu) were removed, as were tokens consisting of a sequence of digits. To maintain consistency with the MeSH data, tokens that were listed for fewer than 4 documents were removed from the vocabulary. The result of this processing was a word-document matrix consisting of 272,926 unique textual tokens and 2,153,769 documents with 175,412,213 word-document pairs. Since some tokens appear multiple times in a document, this matrix was not populated solely with ‘ones’, as was the MeSH matrix, but contained the numbers of times each token appeared in each document. The sum over the entire matrix of occurrences (i.e. the total count of all kept terms in all documents) was 277,008,604. Distributions of MeSH terms and words over documents are available in Supporting Information S1.

### Similarity approaches

This study used five different analytical techniques with two different data sources, and the nine realized combinations (similarity approaches) are shown in Table 1. Four of the five analytical techniques were used with MeSH terms: standard term frequency-inverse document frequency (tf-idf cosine), latent semantic analysis (LSA), a Poisson-based language model for ranking (BM25), and a self-organizing map (SOM). The MeSH-document matrix described above was used as the input to all four of these approaches. Five different analytical techniques were used with title and abstract words: tf-idf cosine, LSA, topic modeling, and two Poisson-based techniques – BM25 and PMRA. The word-document matrix described above was used as the input to all five TA-based similarity approaches. The PMRA approach used ranked lists of PubMed Related Articles (PMRA) downloaded from MEDLINE. Due to the scale of the calculations, and given that our team is comprised of people with expertise in different approaches, the work was distributed as shown in Table 1. The SOM method was applied only to the MeSH-document matrix in consideration of both the computing resources required and the higher dimensionality of the TA-based data.

**Table 1:** Listing of text-based similarity approaches and locations where the similarity calculations were performed.

Similarity approach	Data source	
	MeSH terms	Title/abstract words
tf-idf cosine	tf-idf MeSH (Indiana U.)	tf-idf TA (Indiana U.)
Latent semantic analysis	LSA MeSH (Indiana U.)	LSA TA (Indiana U.)
Topic modeling		Topics TA (UC Irvine)
Self-organizing map	SOM MeSH (SDSU/Indiana U.)	
Poisson-based	BM25 MeSH (Collexis)	BM25 TA (Collexis) PMRA (UC Irvine/SciTech)

The six unique analytical techniques from Table 1 – tf-idf cosine, LSA, topic modeling, SOM, BM25, and PMRA – are each briefly described here. More detailed descriptions of each process step, including methodologies implemented to use these techniques at the scale of two million documents, are available in Supporting Information S1.

#### tf-idf cosine

A standard term frequency-inverse document frequency approach [3] was used. tf-idf coefficients were calculated for each non-zero cell in the matrix as:
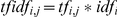



where inverse document frequency is calculated as *idf_i_*  =  *log(D/d_i_)* for each term *i*, *D* is the total number of documents in the corpus, *d* is the number of documents in which term *i* occurs. Term frequency is calculated as *tf_i,j_*  =  *n_i,j_*/∑*n_k,j_*, for each term *i* and document *j* where *n_k,j_* is the number of occurrences of term *k* in document *j*. Document-document similarity values are calculated as the cosine similarity between term vectors as *cos_A,B_*  =  A • B/||A|| ||B|| where A and B are the term vectors for documents A and B.

#### LSA

Latent semantic analysis [28] was introduced in 1990. In its original implementation, singular value decomposition (SVD) was used with a raw term-by-document matrix **X** (containing *D* documents and *N* terms) to compute the singular value matrix **S** using **X = T S D^T^**. **T** is a matrix composed of *N* terms and *k* singular vectors (or concepts onto which the documents load to varying degrees), **S** is a singular value matrix with *k* singular values along its diagonal, and **D** is a reduced document matrix composed of *D* documents and *k* singular vectors. Normalized term-by-document matrices have been used in place of the raw term-by-document matrix in many LSA studies to good effect [29], [30], [31]. We choose to use the tf-idf [32] matrix (from above) as input matrix **X**.

SVD is not practical when the input matrix **X** is large. Instead, we use a Generalized Hebbian Algorithm [33] to approximate matrix **S**. For the LSA TA calculation **S** was limited to the top 100 singular values, and for the LSA MeSH calculation **S** was limited to the top 200 singular values. Once matrix **S** has been calculated, we compute the reduced document matrix **D = (S^−1^ T^T^ X)^T^**. Document-document similarity values are calculated as dot products between pairs of rows in matrix **D**.

#### BM25

BM25, also called Okapi BM25, is a ranking function that is widely used by search engines to rank matching documents according to their relevance to a query [34], [35]. Although rarely used in clustering applications, it is usually used instead of tf-idf for information retrieval, and is very well suited to use with large document sets. The BM25 similarity between a document *q* and another document *d* is calculated as:
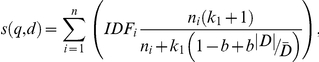



where *n_i_* is the frequency of term *i* in document *d*. Note that *n_i_* = 0 for terms that are in document *q* but not in *d*. Typical values were chosen for the constants *k_1_* and *b* (2.0 and 0.75, respectively). Document length *|D|* was estimated by adding the term frequencies *n_i_* per document. Average document length 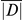 is computed over the entire document set. The IDF value for a particular term *i* was computed as:
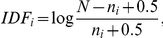



where *N* is the total number of documents in the dataset and *d_i_* is the number of documents containing term *i*. Each individual term in the summation in the first formula is independent of document *q*. For the TA calculation, all IDF scores below 2.0 were discarded, effectively limiting the set of terms used in the calculation to those with *n_i_*<21,324 (i.e., present in less than 0.99% of the documents). For the MeSH calculation, the IDF threshold was set to 1.5 (or *n_i_*<66,020) rather than 2.0.

#### SOM

The self-organizing map (SOM) method is a form of artificial neural network that generates a low-dimensional geometric model from high-dimensional data [36]. The map itself is a grid of neurons, each having a vector corresponding to a position in the term space. Each neuron has a numeric, continuous weight for each of the terms, as opposed to the discrete counts contained in the input vectors. All of the neuron weights are initially randomly seeded. During training, one repeatedly (1) presents individual MeSH-document vectors to the neuron grid and identifies the neuron vector to which it is most similar (using cosine similarity), and then (2) pulls that best-matching neuron and its neighboring neurons even closer towards the input document vector. This adjustment is proportional to the grid distance between the best-matching neuron and its neighbors, within a certain neighborhood diameter. Early during training that diameter will be large, extending across most of the map, while at the later training stages only a small range around the most similar neuron is affected. The effect of the resulting self-organization is that topological structures existing in the high-dimensional input space will tend to be replicated in the low-dimensional (here 2-D) model.

The SOM use in this study aimed for a balance between the amount of geometric/topological distinctions (i.e., number of neurons) and the semantic depth (i.e., number of dimensions). Initial experiments with SOM PAK [37] (a standard implementation) indicated that use of the full set of 23,347 dimensions from the MeSH-by-document dataset was computationally unfeasible. Thus, we reduced the dimensionality of the input data by keeping the 2,300 most frequent MeSH terms, which allowed us to construct a SOM of 75,625 neurons (275×275). The resulting model can itself be the basis of visualization, without involving the document vectors as such (Supporting Information S1).

In order to allow some comparison to the other methods, the full set of MeSH-based document vectors was then mapped on the SOM by assigning each document to the best-matching neuron. Since the number of neurons was roughly double the number of clusters in the other solutions, adjacent neurons containing few documents were combined into clusters until each such cluster contained at least 25 documents. Together with those neurons already containing 25 documents, this resulted in 29,941 clusters partitioning the document set.

#### Topic modeling

The topic model – a recently-developed Bayesian model for text document collections [38] – is considered a state-of-the-art algorithm for extracting semantic structure from text collections. The topic model automatically learns a set of thematic topics (in the form of lists of words) that describe a collection, and assigns a small number of these topics to each and every document in the collection. The topic model evolved from earlier dimensionality reduction techniques such as LSA, and could be considered [39] as a probabilistic version of LSA [40].

Some additional preprocessing was done before the word-document matrix was topic modeled. First, 131 topically uninteresting but frequently occurring words were removed from the data (e.g., ‘study’, ‘result’, etc.). All terms that occurred fewer than 50 times across the entire corpus were also removed. This reduced word-document set retained all 2,153,769 documents, but reduced the number of unique tokens from 272,926 to 65,776. The sum of the word-document triples was 243,724,698 (88% of the original number).

Three separate Gibbs-sampled topic models were learned at the following topic resolutions: T = 500, T = 1000 and T = 2000 topics. These topic models were run for: 1600, 1500 and 1200 iterations (one iteration is one entire sweep through the corpus), respectively. Dirichlet prior hyperparameter settings of β = 0.01 and α = 0.05*N*/(*D* ^.^ *T*) were used, where *N* is the total number of word tokens, *D* is the number of documents and *T* is the number of topics.

From the results of these three models, the top-20 most similar documents for each of the 2,153,769 documents in the corpus were computed. A topic-based similarity metric was calculated, using an equal weighting of the T = 500, T = 1000 and T = 2000 topic models. Specifically, the similarity between documents A and B were calculated as:
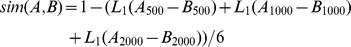



where *L_1_* is the L_1_ norm (the sum of the absolute values of the vector entries), and *A_500_*, etc. are the probabilities for the T = 500, etc. topics of document A.

#### PMRA

The PMRA ranking measure [41] is used to calculate ‘Related Articles’ in the PubMed interface. We consider it the *de facto* standard since it has been through sufficient testing and review to have been accepted by NIH for use in PubMed. PMRA shares a theoretical basis with BM25 in that both use Poisson distributions to model term frequencies. The PMRA implementation used in PubMed uses title and abstract words as well as MeSH headings. In addition, title words are weighted twice as much as abstract words.

We queried PubMed to retrieve the pre-calculated PMRA matches for each document in our corpus. This script did not return PMRA similarity values, but instead returned a rank-ordered list. We post-processed to limit the related articles lists to documents that were in our corpus. Since we did not have actual similarity values, we converted the rank-ordered lists of relationships into similarity values. We created our own proxy for the PMRA similarity as
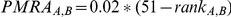



for all articles *B* related by *rank_A,B_* to article *A*. Thus, for any article *A*, the first ranked Related Article was assigned a similarity value of 1.00, the second a similarity value of 0.98, etc. We emphasize that these are not the internal similarity values calculated using the PMRA method (which are unknown to us), but are rather our proxy for these values computed from rank orders. This approach is thus fundamentally different from the other approaches tested.

### Similarity filtering

We applied an additional filtering step to each of the nine similarity matrices to reduce the number of nonzero entries. Similarity matrices with over 25 million similarity pairs (approximately top-12 similar documents for each document) are too large for our clustering routine (a graph layout algorithm) to handle efficiently. Despite the reduction in information from filtering out some less important similarity values, we have previously found that this filtering reduces noise, and actually increases the accuracy of a cluster solution [42], [43].

For this filter we generate a top-n similarity file from each of the larger similarity matrices. The premise behind this is that documents that contribute more overall similarity to the solution space should contribute more similarity pairs to the clustering input. Documents with small similarities should not contribute as much because they are not very similar to any other documents in the corpus. We sum the top-15 similarity values per document, and then scale the number of edges (or pairs) each document should contribute to the similarity file to between 5 and 15 edges using *log(avg(top15 sim))*. Each document thus contributes between 5 and 15 edges to the similarity file. We de-duplicate all (A:B – B:A) pairs for efficiency, and save the top-n similarity files to use as input to the clustering step.

### Clustering

We compute a clustering or partitioning of the document collection using the aforementioned similarity data. Clustering is performed for each similarity file using the detailed multi-step process from [26]. DrL (now called OpenOrd) [44] is a graph layout algorithm that calculates an (x,y) position for each document in a collection using an input set of weighted edges. DrL employs edge cutting, reducing the number of edges by preferentially cutting them based on degree and distance. An average-link clustering routine is then used to assign each document to a cluster based on proximity and remaining edges. This DrL/average-link combination is run 10 separate times with different starting points to generate 10 unique, but highly overlapping solutions. The results are then re-clustered using only those document pairs that are clustered together in at least 4 of the 10 preliminary solutions. Clusters can only be joined together in the final solution by document pairs that are clustered together in 7 of the 10 preliminary solutions. Using this method and criteria the clusters are extremely well defined and one can use single link clustering without experiencing chaining effects. Finally, we require a minimum cluster size of 25 documents; thus, clusters with fewer than 25 documents are merged with the cluster which is most similar (based on similarities between cluster members) until no clusters with fewer than 25 members remain.

This clustering methodology will not necessarily assign all documents to a cluster. If a document is not paired with any other single document in the corpus in at least 4 of the 10 preliminary solutions, it is dropped from the cluster solution. If a document is dropped from the solution, it is an indication that the document could not be assigned to a cluster. If a large fraction of documents are dropped from a particular solution, it is an indication that the similarity approach has a high level of ambiguity. Coverage, or the fraction of documents retained in a cluster solution, is thus an important metric in judging similarity approaches.

### Validation measures

Many studies that compare cluster solutions do so using pre-defined document sets based on expert opinion, such as those used in TREC [14]. Others use the ratio of within-cluster similarity to between-cluster similarity, with higher ratios denoting a better cluster solution [45], often times using the same feature upon which the clustering was based. Given the corpus size used in this study, comparison with expert opinion was not an option. We chose to assess and compare cluster solutions using two different types of validation measures: (1) within-cluster textual coherence based on the Jensen-Shannon divergence, and (2) concentration measures based on grant-to-article linkages indexed in MEDLINE.

#### Textual coherence

We measure textual coherence using the Jensen-Shannon divergence (JSD) [27], which computes the distance between two probability distributions. JSD is calculated for each document from the word probability vector for that document, and from the word probability vector for the cluster in which the document resides as:
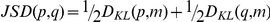



where *m = (p+q)/2*, *p* is the probability of a word in a document, *q* is the probability of the same word in the cluster of documents, and *D_KL_* is the Kullback-Leibler divergence
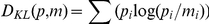



JSD is calculated for each cluster as the average JSD value over all documents in the cluster.

JSD is a divergence measure, meaning that if the documents in a cluster are very different from each other, using different sets of words, the JSD value will be very high. Clusters of documents with similar sets of words – a less diverse set of words – will have a lower divergence. JSD also varies with cluster size – larger clusters will naturally be more divergent than smaller clusters. We normalize by calculating JSD for random clusters of different sizes drawn from the corpus [26]. For example, *JSD(rand)* for cluster size 20 is based on the average JSD of 5,000 clusters of size 20 drawn randomly from the corpus. Coherence is calculated from divergence values for each cluster *i* as:
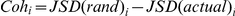



where *JSD(rand)* is the random divergence for the particular cluster size. The average coherence value for an entire cluster solution is then calculated as a weighted average:
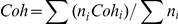



summed over all clusters *i* where *n_i_* is the size of cluster *i*.

Although textual coherence does distinguish between the textual similarity approaches as will be shown below, we note that use of this measure may not be unbiased, simply because the validation inputs (title and abstract words) are not independent of the clustering inputs (title and abstract words or MeSH terms). In addition, we note that articles with titles and abstracts that do not adequately reflect the content of the article are unlikely to be well clustered using any method.

#### Concentration

In addition to textual coherence as a useful measure of cluster quality, we included a second measure to compare cluster solutions. We created a metric based on grant acknowledgements from MEDLINE, using a grant-to-article linkage dataset from a previous study [46].

The premise for using grant-to-article linkages as a metric for measuring the accuracy of a cluster solution is the assumption that the articles acknowledging a single grant should be highly related, and should be concentrated in a cluster solution of the document space. Using this approach, a cluster solution giving a higher concentration of grants is more accurate than one with a lower concentration value. Grant acknowledgements are unrelated to the textual similarity approach and thus provide an independent and unbiased metric for cluster quality.

To measure concentration, we must limit to those grants that can show a concentrated solution. For example, grants that have only produced one article cannot differentiate between cluster solutions. Thus, we limited the grant-to-article linkage set to those grants that have produced a minimum of four articles. The resulting basis set consisted of 571,405 separate links between 262,959 unique articles (over 12% of the corpus) and 43,442 NIH grants.

We calculate two different concentration measures based on grant-to-article linkages: a standard concentration (or Herfindahl) index and precision-recall. The Herfindahl index is calculated for each grant *i* as 
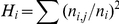



where *n_i,j_* is the number of articles acknowledging grant *i* in cluster *j*, and *n_i_* is the total number of articles acknowledging grant *i*. An overall value for each cluster solution is then calculated as the weighted average over all grants, *H = n_i_ H_i_/(∑n_i_).*

Precision and recall are typically computed using responses to a query where the set of correct responses is known *a priori*. Recall is the fraction of all correct responses that are retrieved by the query, while precision is the fraction of the actual retrieved responses that belong to the set of correct responses. Since our cluster solutions do not consist of queries, we must formulate precision and recall in a different, but analogous, manner. First, we assume that the set of correct responses is the set of 262,959 unique articles linked to grants as mentioned above. We calculate precision and recall by ordering all clusters in a solution by the fraction of correct articles in the cluster, and then calculating the cumulative fractions of correct links (recall) and correct articles (precision) as one proceeds down the list of clusters. Precision decreases as recall increases. A detailed example is given in Supporting Information S1.

The advantage of the Herfindahl index is that it is calculated on a grant-by-grant basis and then averaged over grants, thus ensuring high specificity. The advantage of precision-recall is that it gives curves that show a distribution of metric values. However, since articles referencing multiple grants can appear in the same cluster, precision-recall is a far less specific measure.

## Results

### Characteristics of cluster solutions

**Figure 1:**
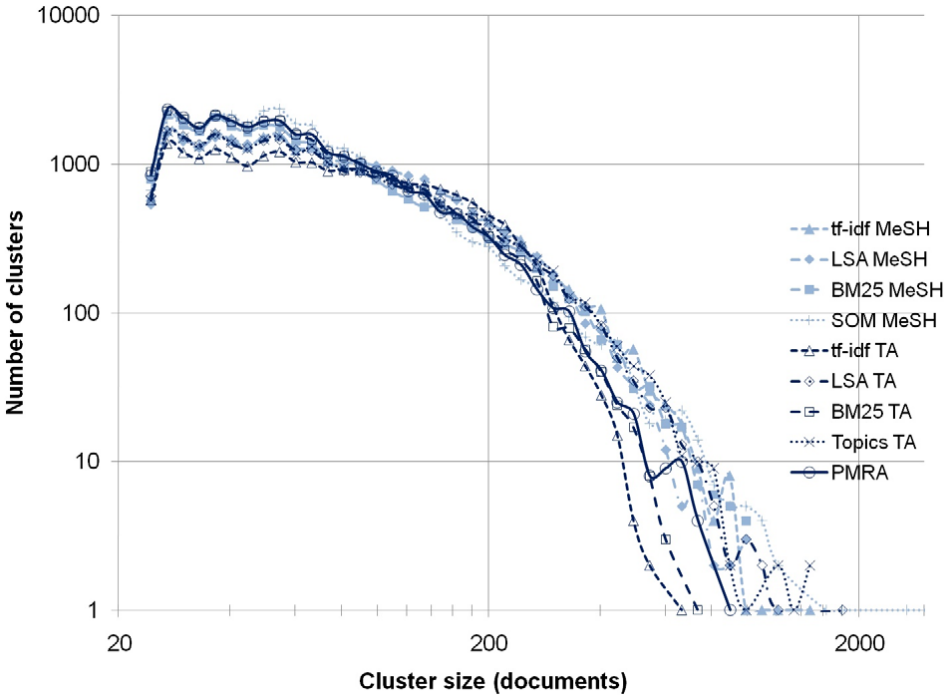
Cluster size distributions for the nine similarity approaches.

Metrics from the cluster solutions from each of the similarity approaches are given in Table 2, while cluster size distributions are shown in Figure 1. Metrics include the numbers of documents that remained in the cluster solution, along with the numbers of clusters and maximum cluster sizes.

**Table 2:** Characteristics of the cluster solutions for the nine similarity approaches.

Approach	# Articles covered	% Coverage	# Clusters	Max Cluster Size
tf-idf MeSH	2,062,642	95.77%	24,708	1517
LSA MeSH	2,115,440	98.22%	25,287	1021
BM25 MeSH	2,011,339	93.39%	26,864	1015
SOM MeSH	2,153,169	99.97%	29,941	3576
tf-idf TA	1,796,349	83.41%	21,388	657
LSA TA	1,958,125	90.92%	23,831	1827
BM25 TA	2,022,694	93.91%	28,858	764
Topics TA	2,033,221	94.40%	24,163	1422
PMRA	2,029,564	94.23%	28,963	921

The clustering results lead to several observations. First, the tf-idf TA approach has the lowest coverage (fraction of the corpus that was clustered) at 83.4%. This measure also had the largest number of similarities in its input file (24.3 million) of all of the measures tested. These two factors – the large number of input similarities and the low coverage – are likely related. Although the filtering method used to generate the top-n similarity files for this measure was the same as that used for the other text-based similarities, the distribution of similarities (leading to the top-n assignment) was quite different, and gave rise to a larger similarity file. We speculate that this is due to slight variations in similarity between document sets arising from the high end of the word-document distribution (those words that occur in a very large fraction of documents). Other TA approaches (BM25 and Topics) both applied additional processing to the matrix that would have mitigated such behavior. The SOM MeSH approach had extremely high coverage; all but just a few hundred documents in the set were assigned to a cluster.

Second, the numbers of clusters from nearly all of the approaches are in a similar range (24,000 – 30,000 clusters), and thus are suitable for the comparisons that will be reported in a subsequent section. The tf-idf TA approach has fewer clusters to go with its lower coverage, but even this is within an acceptable range for evaluation.

### Accuracies of cluster solutions

#### Coherence

**Figure 2:**
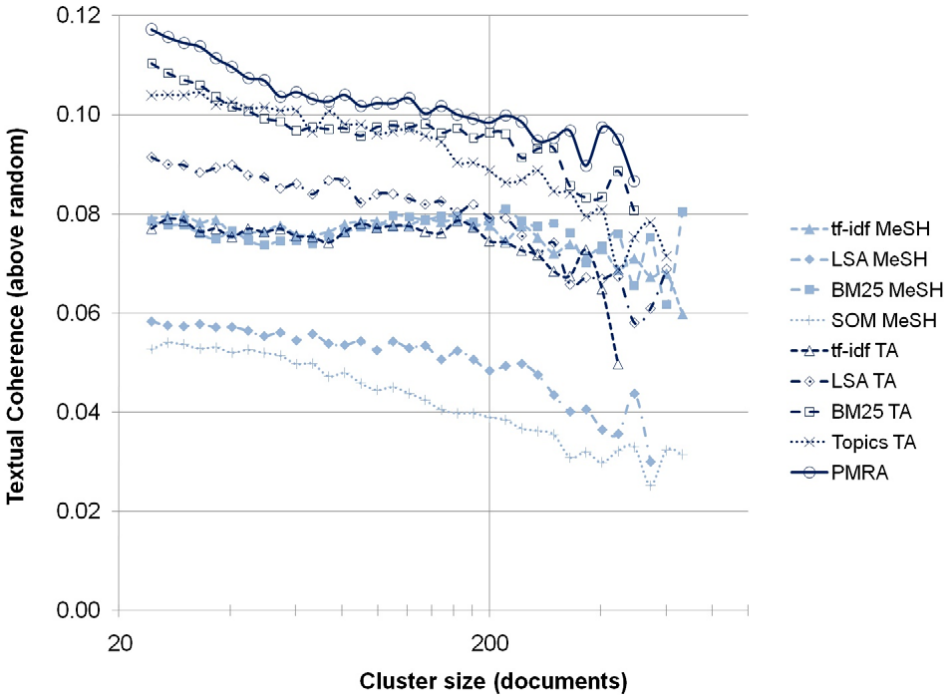
Textual coherence values by cluster size for the nine similarity approaches. Coherence is a measure of cluster quality. A higher value of coherence indicates a higher degree of textual similarity between the titles and abstracts within a cluster than does a lower value of coherence. Data are shown for cluster size bins of at least 15 clusters.

Textual coherence distributions by cluster size for the nine cluster solutions are shown in Figure 2. Only cluster bins with 15 or more measurements are shown. Most of the curves show a similar trend – textual coherence decreases slightly with increasing cluster size. Two of the MeSH-based measures (tf-idf and BM25) have relatively flat distributions. The PMRA measure has the highest coherence values over the entire range of cluster sizes, followed closely by the BM25 TA measure.

Comparison of the coherence values from the different cluster solutions leads to some very interesting observations about the different similarity approaches:

- The BM25 TA approach significantly outperformed the tf-idf TA approach, even though it was based on the same initial word-document matrix. The BM25 TA calculation differed from the tf-idf TA calculation in two major ways: 1) it limited the word set to those that occur in less than 0.99% of the documents instead of using the full word-document matrix, and 2) it used the BM25 similarity approach in place of the standard tf-idf. The effect of the first change (truncating the word distribution) was to remove a large amount of noise from the solution space. The effect of the second change (BM25) was to use a superior similarity approach, as has been established in the literature. Combined, these two changes had an enormous positive effect on the accuracy of the cluster solution.
- The PMRA approach performed slightly better than the BM25 TA approach. The PMRA approach differs from the BM25 approach in three main ways: 1) it does not remove all high frequency words, but rather removes a set of 132 high frequency, low content words, 2) it counts words in the title twice rather than once and also uses MeSH terms, 3) it uses the PMRA similarity measure rather than the BM25 measure. The original work by Lin and Wilbur showed that the PMRA measure slightly outperformed BM25 over a range of conditions [41]. Given these differences, it is likely that the overall difference in performance between these two approaches is in the use of the PMRA measure over the BM25 measure, and the double-counting of title words.
- The topics TA approach also outperformed the tf-idf TA approach, but did not do nearly as well as the BM25 TA or PMRA approaches. The topics TA method was similar to the PMRA approach in that it removed 132 high frequency, low content words. However, it also removed all words occurring in fewer than 50 documents. The major difference between this approach and the BM25 TA and PMRA approaches is in the use of the topic modeling algorithm rather than the BM25 or PMRA similarity measures. It appears that BM25 and PMRA do better than topic modeling for generating a fine grained cluster solution of a large portion of the scientific literature.
- A comparison of the BM25 MeSH and BM25 TA results shows that titles and abstracts are far superior to MeSH terms as a basis for clustering of documents. In addition, a comparison of the co-Word MeSH and BM25 MeSH results suggests that the application of the BM25 algorithm (as opposed to tf-idf) on MeSH terms makes very little difference in the outcome. The use of the BM25 algorithm has a far greater effect when used with words extracted from titles and abstracts than with MeSH terms, likely because so many more tokens are available per article.

#### Concentration

**Figure 3:**
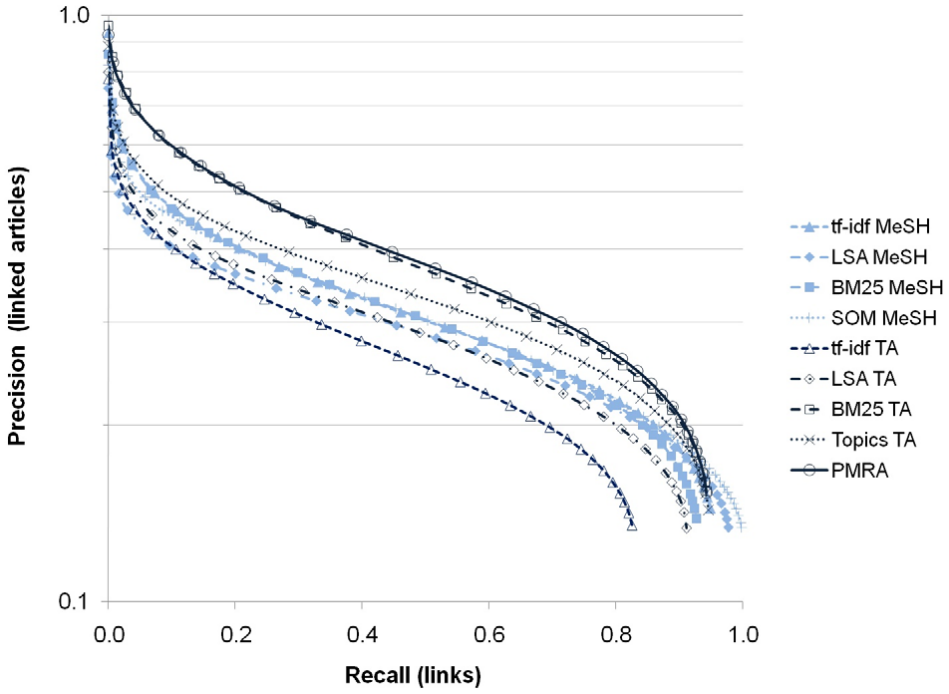
Precision-recall curves for each cluster solution based on grant-to-article linkages. To calculate precision-recall, clusters are first ordered by the fraction of articles referencing an NIH grant. Precision is the cumulative fraction of articles referencing the NIH grants, while recall is the cumulative fraction of articles in the cluster solution.

Precision-recall curves were calculated for each cluster solution using the set of grant-to-article linkages mentioned above, and are shown in Figure 3. A higher precision value denotes a higher concentration of papers referencing the set of grants. The PMRA and BM25 TA curves are significantly higher than the other curves, with the PMRA solution giving slightly higher precision than the BM25 TA solution. Curves from the MeSH-based solutions have higher recall at the end, but only because they all have greater coverage than the PMRA and TA-based approaches (Table 2), and thus cover a larger fraction of the 571,405 links overall.

Precision at 80% recall (Pr80) and the maximum value of F1 (the harmonic mean of precision and recall, calculated as 2*P*R/(P+R)) are reported for each cluster solution in Table 3. The maximum F1 values for each solution occur at recall values near 0.60 for each of the solutions. Herfindahl index values for the solutions are also included in Table 3. The rank orders of the nine approaches across the different concentration measures listed in Table 3 are relatively constant. For example, PMRA ranks first and BM25 TA ranks second in all measures, and topic modeling ranks third in all but one measure (Herfindahl), where it ranks fourth. The approach with the widest variation in concentration measures was the SOM, which ranked last in Herfindahl, and fifth on average for the precision-recall measures. This suggests that for the SOM approach, there is far more mixing of different grants (at lower individual concentrations) in individual clusters than occurs for the other approaches and a lower Herfindahl value.

**Table 3:** Summary of concentration results for the nine similarity approaches.

Approach	Herfindahl	Max(F1)	Pr80
tf-idf MeSH	0.1631	0.3790	0.2216
LSA MeSH	0.1124	0.3662	0.2127
BM25 MeSH	0.1570	0.3791	0.2167
SOM MeSH	0.1106	0.3796	0.2203
tf-idf TA	0.1299	0.3344	0.1571
LSA TA	0.1255	0.3646	0.2003
BM25 TA	0.2393	0.4281	0.2578
Topics TA	0.1584	0.4011	0.2379
PMRA	0.2410	0.4350	0.2637

## Discussion

There are many dimensions to determining the most accurate similarity approach for clustering a set of over two million biomedical documents. Although we have already discussed coverage, coherence and concentration metrics, it is also useful to consider the computational cost of the different approaches. Table 4 compiles some results from previous tables and adds computational cost and average coherence values.

**Table 4:** Summary of results and decrements in metrics with respect to the PMRA values.

Method	Comp Cost	Coherence	Herf	Pr80	Coh vs. PMRA	Herf vs. PMRA	Pr80 vs. PMRA
tf-idf MeSH	Medium	0.0764	0.1631	0.2216	−26.3%	−32.3%	−16.0%
LSA MeSH	Very high	0.0519	0.1124	0.2127	−49.1%	−53.4%	−19.3%
BM25 MeSH	Medium	0.0765	0.1570	0.2167	−26.2%	−34.9%	−17.8%
SOM MeSH	Very high	0.0452	0.1106	0.2203	−56.4%	−54.1%	−16.5%
tf-idf TA	High	0.0758	0.1299	0.1571	−26.8%	−46.1%	−40.4%
LSA TA	Very high	0.0815	0.1255	0.2003	−21.3%	−47.9%	−24.0%
BM25 TA	High	0.0980	0.2393	0.2578	−5.4%	−0.7%	−2.2%
Topics TA	High	0.0937	0.1584	0.2379	−9.6%	−34.3%	−9.8%
PMRA	Low	0.1036	0.2410	0.2637			

A comparison of the nine similarity approaches shows that there is a range of computational costs; MeSH-based approaches are less computationally expensive than TA-based approaches because there are far fewer tokens to consider. The LSA method needs far more computation than do the simpler tf-idf and BM25 approaches. The neural network training portion of the SOM method, as applied here, does as well, though this was largely due to the goal of a detailed mapping of the document space. The PMRA approach, if document-document similarities were calculated from scratch, would have a similar computational cost to the BM25 approach, but we list the computational cost for PMRA as low because the coefficients are already calculated by PubMed, and thus do not need to be recalculated. The PMRA and BM25 TA approaches have the highest values on the coherence and concentration metrics, and provide sufficient coverage (94%, Table 2) to make them the most attractive text-based approaches for clustering extremely large document sets.

Although the PMRA approach performed best on all accuracy metrics, we note (as was done earlier) that this study used an estimated similarity for PMRA based on rank order rather than the actual similarity values. We do not know if the actual PMRA similarity values would have performed better or worse than the estimated similarities, and thus our conclusions about PMRA are not definitive. However, we do conclude that PMRA with estimated similarities did perform best among all of the approaches considered here.

For the LSA, SOM, and topic modeling approaches, there are many variants in terms of parameter space that can be chosen. This study only investigated one variant in each case. For LSA, the matrix reduction approach and the number of factors are both variables. The numbers of factors used in this study were less (100 and 200) than what is considered typical (300–500 factors) for most studies. It is thus quite possible that the LSA results could be improved if more factors were used. However, increasing the number of factors would also increase the computational cost.

Regarding self-organizing maps, the number of input dimensions is a key consideration. Attempting to use all of the original input tokens proved to be computationally unfeasible, for both MeSH-based and TA-based data sets (only the former was ultimately implemented), when combined with the simultaneous goal of a high-resolution 2-D model of the input space. Given the filtering of MeSH data down to the 2,300 most prevalent (and thus least specific) terms, one could not have expected to produce the most accurate clustering at fine scale, as measured in this study. Meaningful reduction of dimensions is a key strategy for future work – for example, the topics resulting from topic modeling could be used as input dimensions for SOM training. The power of the SOM method to drive engaging and meaningful visualizations of top- and medium-scale structures was demonstrated in the study.

For the topic modeling approach, fine tuning of the approach might increase its accuracy. One obvious step for future study is to compute a similarity measure that blends BM25 and topic model distance.

In this study we sought to answer the question as to which text-based similarity approach would generate the most accurate cluster solution of a large set of biomedical literature. We did this using a large corpus (2.15 million MEDLINE articles) and generated cluster solutions using nine different text-based approaches.

Three different accuracy measures were used to compare the results from the nine approaches. The PMRA approach performed best on all measures, followed closely by the BM25 TA approach. This study used a corpus of over two million documents, a set two orders of magnitude larger than those used in previous studies. As mentioned in the introduction, previous studies at small scale have shown conflicting results that are likely field-specific. However, given the scale of this study and the large degree of separation between the PMRA and BM25 approaches and the other approaches (Table 4), we consider these results to be relatively robust.

**Figure 4:**
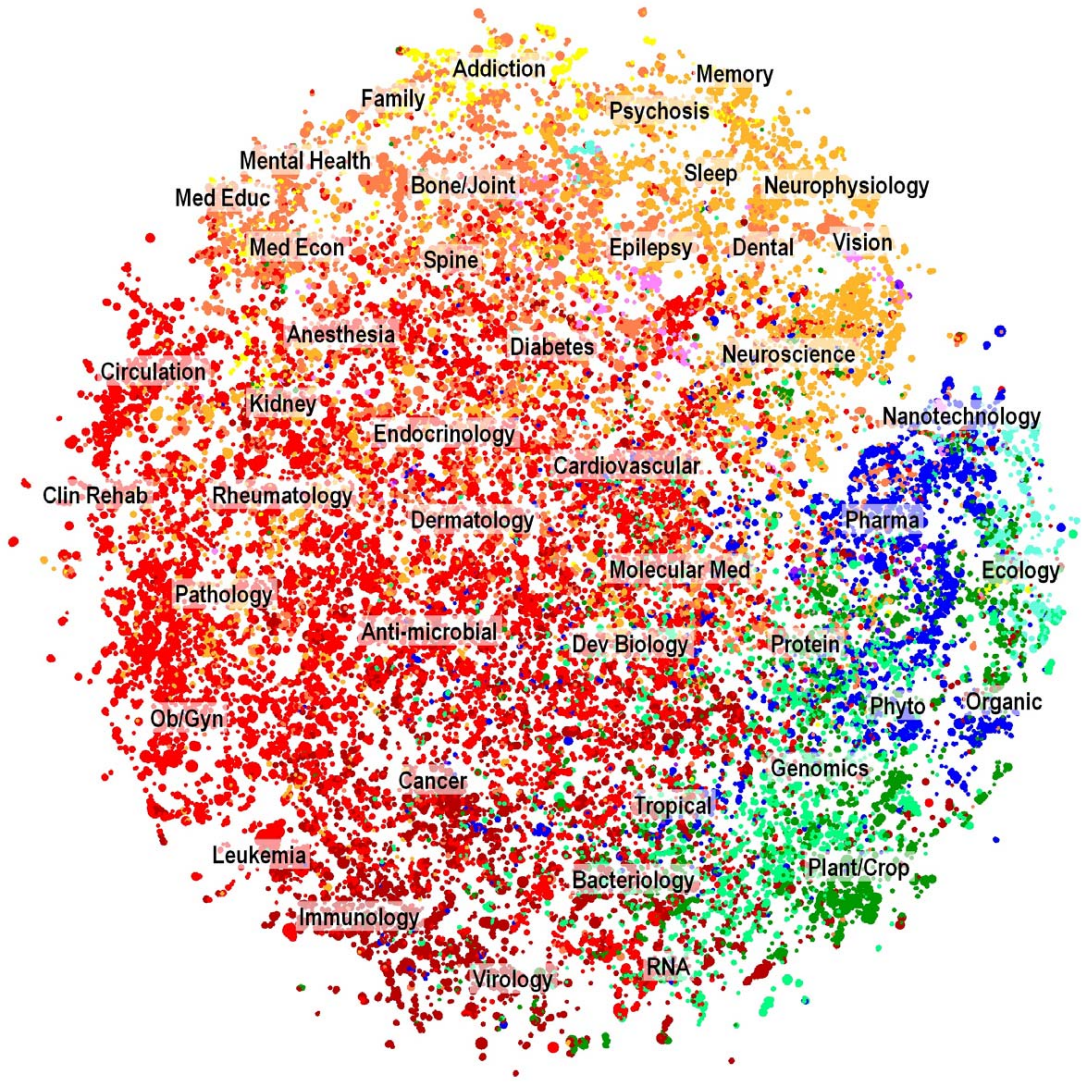
Two-dimensional map of the PMRA cluster solution, representing nearly 29,000 clusters and over two million articles. The map was generated with cluster-to-cluster similarity values using the DrL graph layout routine [44]. Color legend: Chemistry (blue), Engineering (cyan), Biology (green), Biotechnology (teal), Infectious Disease (brick red), Medical Specialties (red), Health Services (peach), Brain (orange), Social Sciences (yellow), Computer Sciences (pink).

As an example of how results from this study could be used in a practical manner, Figure 4 shows a two-dimensional map of the nearly 29,000 clusters in the PMRA solution. Positions for each cluster were calculated using DrL with cluster-to-cluster similarity values (summed from the document-document similarity values) as input. Clusters with related content are proximate to each other on the map. Each cluster is represented by a colored dot. Colors are based on journal distributions by cluster using a color scheme derived from our previous work mapping journals to disciplines ([47], Fig. 2). Labels were added manually based on inspection of terms and journal distributions associated with the clusters in different portions of the map. These labels are not intended to be prescriptive, but merely show in general where various disciplines and concepts represented in MEDLINE are centered in the map space.

It is not our purpose to explore this map in detail here, but rather to mention how such a map could be used. This map gives a visual overview of the structure and content of MEDLINE, thus providing a high degree of context as well as content. A map such as this could easily be used to display the results of traditional queries to MEDLINE. With an appropriate interface, areas with concentrated results could be explored more closely. Clusters with large numbers of hits would be natural areas to explore since each cluster contains a highly focused set of related documents. We recommend the use of a visual interface such as this in conjunction with MEDLINE.

Finally, we note that most of the data from this study, the list of PMID, titles and abstracts, MeSH-document and word-document matrices, similarity files, cluster solutions, and coherence results are available for download at http://sts.cns.iu.edu. We invite others to use these data to make further comparisons; they should be very suitable for the development, testing, and comparison of similarity approaches, clustering algorithms and accuracy measurement approaches.

## Supporting Information

Supporting Information S1 **Supplementary methods, figures, and tables.**
(DOC)
