# Supplementary material for: Clustering More than Two Million Biomedical Publications: Comparing the Accuracies of Nine Text-Based Similarity Approaches
Source: PLoS One. 2011 Mar 17;6(3):e18029. doi: 10.1371/journal.pone.0018029 (PMC3060097; doi:10.1371/journal.pone.0018029)
Supplement: Supporting Information S1 — Supplementary methods, figures, and tables. (DOC) [file pone.0018029.s001.doc]

Supplementary Information on Data and Methods for
“Clustering More Than Two Million Biomedical Publications: Comparing the Accuracies of Nine Text-Based Similarity Approaches”

Kevin W. Boyack^a^, David Newman^b^, Russell J. Duhon^c^, Richard Klavans^d^, Michael Patek^d^, Joseph R. Biberstine^c^, Bob Schijvenaars^e^, André Skupin^f^, Nianli Ma^c^ & Katy Börner^c^

^a^ SciTech Strategies, Inc., Albuquerque, NM USA ([kboyack@mapofscience.com](mailto:kboyack@mapofscience.com))
^b^ University of California, Irvine, Irvine, CA USA and NICTA Australia
^c^ Cyberinfrastructure for Network Science Center, SLIS, Indiana University, Bloomington, IN USA
^d^ SciTech Strategies, Inc., Berwyn, PA USA
^e^ Collexis, Inc., Geldermalsen, The Netherlands
^f^ San Diego State University, San Diego, CA USA

**Data and Methods**

***Study corpus***

The purpose of the full study that was performed for NIH was to find the most accurate science mapping solution on a very large corpus of biomedical literature – in essence, to determine how to most accurately map all of the medical sciences literature. As mentioned previously, even though we only report on text-based approaches here, the full study compared text-based, citation-based, and hybrid text-citation approaches. A study corpus was needed that would be large enough to provide definitive results, and that would not be biased toward either text- or citation-based approaches. We thus generated a corpus for which we had both sufficient text and citation information for all articles.

Given that NIH hosts the MEDLINE database, its contents are an implicit definition of what the NIH considers to be medical science. We thus felt it best to base our study corpus on MEDLINE, and to add citation data as needed. Scopus records were matched to MEDLINE records to generate a set of records with both textual and citation information. This set of records was then limited to those articles published from 2004-2008 that contained abstracts, at least 5 MeSH terms, and at least 5 references in their bibliographies, resulting in a study corpus of 2,153,769 unique scientific articles. The detailed process used to generate this corpus, along with matching statistics, is available in .

Numbers of documents by year, using different limitations, are given in Table 1. It is interesting to examine these numbers. Only 81.7% of the MEDLINE records in Scopus have 5 or more references, only 83.5% of the records have abstracts, and only 74.9% of records have both. This suggests that if one wants a map with full coverage, a hybrid approach would be necessary – otherwise, a citation-based map would be missing around 18% of documents, and a text-based map would be missing 16%. For the study corpus, it was decided to keep those documents with an abstract, at least five and fewer than 400 references, and at least 5 MeSH terms. The final numbers of articles by year that met these criteria are listed in the final column of Table 1. We also checked our matching process by accessing the PMIDs as indexed in the Scopus raw data, and found that our matching process had an accuracy of > 99.9% .

**Table 1**. Numbers of documents by year using different limitations.

| **Year** | **MEDLINE** | **In Scopus** | **A** | **R** | **A or R** | **A and R** | **Final** |
| --- | --- | --- | --- | --- | --- | --- | --- |
| 2004 | 575,938 | 553,743 | 454,023 | 436,421 | 489,545 | 400,899 | 389,353 |
| 2005 | 603,166 | 579,359 | 480,477 | 469,777 | 517,773 | 432,481 | 420,059 |
| 2006 | 626,895 | 605,734 | 504,747 | 498,328 | 547,663 | 455,412 | 442,743 |
| 2007 | 644,457 | 620,386 | 523,805 | 520,196 | 566,781 | 477,220 | 464,479 |
| 2008 | 650,069 | 597,839 | 506,852 | 490,034 | 547,110 | 449,776 | 437,135 |
| Total | 3,100,525 | 2,957,061 | 2,469,504 | 2,414,756 | 2,668,872 | 2,215,788 | 2,153,769 |

A – has an abstract; R – has > 5 references

***Similarity approaches***

Nine different text-based maps, as listed in Table 2, were generated using the textual information associated with our corpus of 2,153,769 documents. Four of the maps were based on MeSH terms and four of the maps were based on titles and abstracts (TA). The final approach, the PubMed related articles (pmra) approach, was added because it is used on the PubMed website, already has the PubMed stamp of approval, and thus exists as a *de facto* standard.

Table 2. Listing of the text-based similarity approaches used in this study.

| **Similarity basis** | **Technique** | **Approach name** | **Location** |
| --- | --- | --- | --- |
| MeSH terms | Co-word (TFIDF) | Co-word MeSH | IU |
| MeSH terms | Latent semantic analysis | LSA MeSH | IU |
| MeSH terms | Self-organizing map | SOM MeSH | SDSU/IU |
| MeSH terms | bm25 algorithm | bm25 MeSH | Collexis |
| Titles / abstracts | Co-word (TFIDF) | Co-word TA | IU |
| Titles / abstracts | Latent semantic analysis | LSA TA | IU |
| Titles / abstracts | Topic modeling | Topics TA | UC Irvine |
| Titles / abstracts | bm25 algorithm | bm25 TA | Collexis |
| Titles / abstracts / MeSH | PubMed related articles | pmra | UC Irvine/STS |

Although each of MeSH and TA-based approaches require different processing, they each start from common term-document matrices. Two different term-document matrices were extracted from the MEDLINE corpus: a MeSH-document matrix and a term-document matrix, where terms for the latter were parsed from the titles and abstracts of the documents. The MeSH-document matrix was used as the input for all four of the MeSH-based approaches listed in Table 2, while the term-document matrix was used for all four of the TA-based approaches. The following describes the pre-processing that was required to generate the MeSH-document and term-document matrices.

***MeSH preprocessing***

PMIDs and associated MeSH terms (without qualifiers) were extracted from Indiana University’s Scholarly Database (SDB) version of MEDLINE for all documents in the corpus. Whitespace was stripped off each end of each term, and all leading ‘*’ characters were also stripped. No tokenization of MeSH terms was required because they are all standardized indexing terms. The numbers of articles and fraction of articles in the corpus associated with each MeSH term were then computed to determine if any thresholding of terms would be needed.

MeSH terms were limited in the following two ways:

- All terms that are not Class 1 descriptors per the 2009 MeSH data were removed from the set (<http://mbr.nlm.nih.gov/Download/2009/MeSH/README>). This had the effect of removing the Class 3 (Check Tags) and Class 4 (Geographical Locations) terms, which have little or nothing to do with content. Class 2 terms are article types, and are not listed with the MeSH terms.
- To maintain consistency with the reference data, all MeSH terms that were listed for fewer than 4 documents were removed from the set. The upper end of the distribution was left intact because many of the researchers on this team felt that those terms occurring in many documents (e.g., the MeSH term *Humans* is indexed for 66% of the documents) would be useful to the calculation of similarities. The final distribution of MeSH terms thus identified is shown in Figure 1.


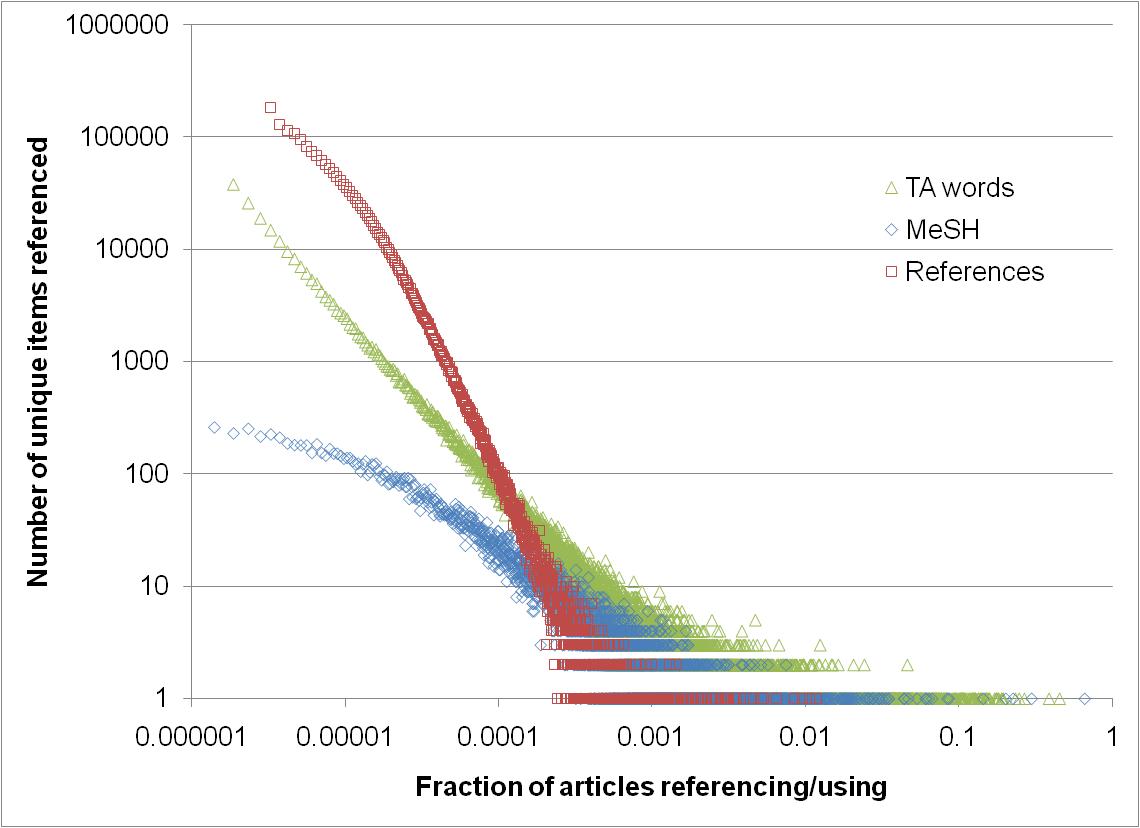


**Figure 1. Final distribution of MeSH terms, words from titles and abstracts, and references within the study corpus.**

The final MeSH-document matrix is based on the MeSH term list associated with the final distribution in Figure 1; the number of unique MeSH terms in the final MeSH-document matrix was 23,347 with a total number of 25,901,212 descriptors (MeSH terms) occurring in 2,153,769 documents.

***Title/abstract (TA) preprocessing***

PMIDs and associated titles and abstracts were extracted from the SDB version of MEDLINE for all documents in the corpus. The extracted text was then parsed using the following process designed to mimic standard stemmers and tokenizers.

All punctuation characters except apostrophes were removed from the text, and replaced with a single space each. The resulting text was converted to all lower case and split on whitespace, leaving only tokens with no whitespace in them, and no empty tokens. Then, each token with a contraction ending in 'll, 're, 've, 's, 'd, or n't was separated into a root and a contraction. The contraction portions were removed; all of the contraction suffixes are forms of words found on standard stopword lists or are possessive forms of other words.

All tokens appearing on our stopword list (which is a combination of an official MEDLINE stopword list of 132 words, and a second list of 300+ words commonly used at NIH and provided by David Newman, UC Irvine) were then removed, as were tokens consisting of a sequence of digits.

Tokens were then further limited using the same methodology applied to MeSH terms; all tokens that were listed for fewer than 4 documents were removed from the set. The final distribution of tokens from titles and abstracts thus identified is shown in Figure 1. The number of unique tokens in the final word-document matrix is thus 272,926 with a total number of 175,412,213 tokens occurring in the 2,153,769 documents. The total number of individual token occurrences is 277,008,604; this number differs from the previous number in that some tokens occur multiple times in the same document. Thus, there are on average 128.6 individual title/abstract-based token occurrences associated with each document.

***Textual similarity techniques***

Among the nine approaches listed in Table 2, there are six separate analytical techniques that were tested. These were:

- Co-word (TFIDF) analysis, also known by labels such as term co-occurrence,
- Latent semantic analysis (LSA),
- Topic modeling (Topics) based on Latent Dirichlet Allocation,
- Self-organizing maps (SOM),
- bm25 similarity measure (bm25), and
- (PubMed related articles (pmra).

Given the scale of these calculations with a set of over two million documents, generation of the pair-wise similarity values from the initial MeSH-document and term-document matrices was done at different locations, as listed in Table 2. In nearly all cases existing codes were rewritten or tuned to be able to efficiently calculate on the order of 2 trillion pair-wise similarities (roughly the number of cells in the upper half of a 2M x 2M matrix).

*Co-word analysis using TFIDF:* The co-word analysis steps described here were run in turn on both the MeSH-document and term-document matrices. To reduce computation time, all terms and documents were replaced with integers that uniquely identified them. This allows later calculations to only store a single integer per document or term, instead of having to store an entire string of characters.

Before running the similarity calculations, tfidf (term frequency, inverse document frequency) was performed on the input data as follows:

- For each term *i*, inverse document frequency was calculated as *idf_i_* = *log(D/d_i_)*, where *D* is the total number of documents in the entire corpus and *d* is the number of documents in which term *i* occurs,
- For each term *i* and document *j*, term frequency was calculated as *tf_i,j_* = *n_i,j_* / ∑*(n_k,j_)*, where *n* is the number of occurrences of term *k* in document *j*; the denominator sums over all terms *k* in document *j*.
- Calculate tfidf as *tfidf_i,j_* = *tf_i,j_* * *idf_i_* .
- The resulting matrices of tfidf coefficients were stored for further use.

Cosine similarities between pairs of document vectors were then calculated using standard equations. However, the code to generate these cosine values was modified to run in the following manner due to the extremely large size of the data:

- The data were split into many subsets of a few thousand documents each. Computations covering different ranges of documents were done in parallel. For each subset –
- *tfidf* coefficients for the range of documents needing similarities computed, and all documents they will be compared to, were loaded into memory,
- For each document, a sorted list of non-zero terms was retained, along with their associated *tfidf* coefficients,
- For each document A, the Euclidean norm ||A|| of the term vector was calculated as ||A|| = *√(∑ (tfidf_i_^2^))* over all terms *i*.
- For each document an empty min-heap was prepared to hold the top 50 values,
- For each document A, the cosine similarity between it and all documents B with which it is to be compared was calculated as cosine = A • B / ||A|| ||B||, where the numerator is the dot product of the *tfidf* vectors associated with documents A and B, and the denominator consists of the Euclidean norms. An efficient way to calculate the dot product of the two very sparse vectors is to compute the intersection of the sorted term lists for each document, then multiply and sum only the *tfidf* coefficients of the intersecting elements,
- While computing the cosine similarities for any document A, the top 50 list is effectively managed by loading the first 50 similarities into the min-heap sorted by descending cosine value, and then inserting subsequently calculated values into the appropriate location in the heap if they are large enough. Each time a new value is inserted it pushes the bottom value out of the heap.
- Once all similarities have been calculated for document A, they are written out.

When parsing out the cosine calculation to multiple processes and processors, the MeSH similarity code uses all documents B with each document A. However, the TA calculation was much larger (272,926 TA tokens vs. 23,347 MeSH tokens); thus all documents B could not be placed in the same calculation for any given document A due to memory constraints. Instead, each run of the code uses a large chunk of the total data as the documents to be compared to. This does not add much overhead compared to the having the entire data set in memory. Due to the specific constraints of the computers used to run the calculation, seventeen chunks were used. For the MeSH similarities, subsets of 20,000 documents A were placed in each batch, while for the title/abstract similarities subsets of 5,000 documents A were used. Note that these numbers are specific to this corpus and IU hardware configuration and would need to be adjusted for other data sets or hardware. The target should be, absent external constraints, to allow as many processes to fit in memory simultaneously as there are processing cores on the computers being run on.

Although this particular calculation (the TA cosine similarities) required significant computing capabilities, the IU computers and code were able to run the full calculation in just five calendar days. However, given the parallel nature of the calculation, this translated to over 310 days of processing time on IU’s Quarry supercomputer. This equates to roughly 75,000 similarities per second per processor, or about 4.5 million similarities per second across all the processes running in parallel. Considering the high dimensionality of the data, we consider this to be a very high throughput.

*Latent semantic analysis (LSA):* LSA is a technique for reducing the dimensionality of a term-document matrix by finding lower rank matrices that together approximate the information in the full matrix . It works by minimizing the sum of the squares of differences between the entries in the original matrix and the approximations of those values. Its advantages over traditional vector space techniques include the ability to account for synonyms.

LSA typically uses the following process. First, normalized term-document coefficients, such as those obtained with either *tfidf* or log-entropy weighting, are the typical starting point for an LSA calculation. A singular value decomposition (SVD) calculation is then performed on the entire matrix to compute the singular value matrix **S** using **X = T S D^T^** , where **X** is the original *tfidf* matrix with *D* documents and *N* terms, **T** is the ‘term’ matrix composed of *N* terms and *k* singular vectors (or concepts onto which the documents load to varying degrees), **S** is the singular value matrix with *k* singular values along its diagonal, and **D** is the reduced document matrix composed of *D* documents and *k* singular vectors. *k* is typically on the order of 300-500, and is thus much smaller than *N*. The representation of the reduced matrix **D** is thus much smaller than the original matrix **X**, and much easier to manipulate further. Similarities between document pairs are then typically computed as cosines between rows of the reduced matrix **D** using the dot product between rows.

The use of LSA, besides greatly reducing the size of the matrix, has been shown to lead to similarities between documents that are better at satisfying human expectations than do typical co-word approaches. Although SVD is the most standard LSA approach used to calculate (or approximate) the singular value matrix **S**, there are many other different mathematical techniques that can be used in its place. Such is the case for this calculation; SVD is not always practical at the scale of 2 million documents.

The method used to calculate **S** in this study is based on the Generalized Hebbian Algorithm – GHA [[1]](#footnote-2). GHA LSA approximates the top concepts of the LSA (the singular values of the **S** matrix) one at a time, with tunable parameters governing desired convergence. Since only the top concepts are desired, this is much faster than approaches that calculate all of the LSA concepts simultaneously. While it does not fully minimize the error, the eventual output is only in terms of similarities, and only the highest similarities. Thus, provided the documents have similar relative loadings on the GHA LSA vectors towards the extremes, failing to completely minimize the error described above will have little or no effect on the results. As such, parameters were chosen that provided acceptable run times, and the first few vectors were compared against more strict approximations to verify that this assumption was reasonable. Specifically, the *tfidf* matrices mentioned above for both the MeSH-document and term-document cases were used as the starting points for our LSA calculations, and GHA LSA was run with a convergence length (the parameter that governs how exactly vectors are approximated) of 1,000,000 for the MeSH data, retaining the first 200 concepts, and a convergence length of 10,000 for the TA data, retaining the first 100 concepts.

The output of GHA LSA is the diagonal matrix of singular values **S** and the *N* by *k* term matrix **T**. From this, it is possible to quickly compute the document matrix **D** by multiplying the inverse of the singular value matrix by the transpose of the term-concept matrix by the transpose of the document-term matrix, and taking the transpose of the result. The code described above to calculate cosine similarities was used with the values of matrix **D** to compute the top 50 cosine similarities for each document.[[2]](#footnote-3)

*BM25:* BM25 is a well-known ranking function used in information retrieval . Although not widely used in the science mapping community, since its application is that of scoring a query with respect to a set of documents, it is very suitable for use as a document similarity measure for mapping or partitioning documents.

The specific formula used to compute the similarity between a document *q = (w_1_, …, w_n_)* and another document *d* was:


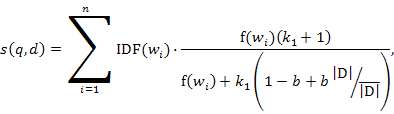


where *f(w_i_)* is the frequency of word *w_i_* in document *d*. Note that *f(w_i_)* = 0 for words that are in document *q* but not in *d*. Typical values were chosen for the constants *k1* and *b* (2.0 and 0.75, respectively). Document length *|D|* was estimated by adding the word frequencies *w_i_* per article. The average document length
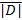
 was computed over the entire document set. The IDF value for a particular word *w_i_* was computed using:


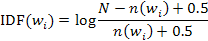


where *N* is the total number of documents in the dataset and *n(w_i_)* is the number of documents containing word *w_i_*. Note that each individual term in the summation in the first formula is independent of document *q*. Hence these were computed first and to keep computing time within acceptable limits, all scores below 2.0 were discarded. (Note, this threshold of IDF > 2.0 acts to limit the word set to words where *n(w_i_)* < 21,324, or words that occur in less than 0.99% of the documents).

For the MeSH terms a slightly different filter was applied to keep computation time acceptable since term frequencies are not available for MeSH terms. Therefore, scores for individual terms follow a different distribution. Scores below 1.5 were discarded, with the exception of the terms of two documents that only contained terms with a score < 1.5.

*Self-organizing maps (SOM):* The self-organizing map (SOM) method is a form of artificial neural network that generates a low-dimensional geometric model from high-dimensional data . The map itself is a grid of neurons, each having a vector corresponding to a position in the term space. Each neuron has a numeric, continuous weight for each of the terms, as opposed to the discrete counts contained in the input vectors. All of the neuron weights are initially randomly seeded. During training, one repeatedly (1) presents individual MeSH-document vectors to neuron grid and identifies the neuron vector to which it is most similar (using cosine similarity), and then (2) pulls that best-matching neuron and its neighboring neurons even closer towards the input document vector. This adjustment is proportional to the grid distance between the best-matching neuron and its neighbors, within a certain neighborhood diameter. Early during training, that diameter will be large, extending across most of the map, while at the later training stages only a small range around the most similar neuron is affected. The effect of the resulting self-organization is that topological structures existing in the high-dimensional input space will tend to be replicated in the low-dimensional (here 2-D) model.

The SOM portion of this study focused foremost on generating a detailed *mapping* of the document space, with the ultimate goal being the creation of useful visualizations of the medical knowledge domain. That is on one hand the single most important reason for the generation of a two-dimensional model of the high-dimensional input space and on the other hand it explains our goal of attempting to use as many neurons as computationally feasible. This is different from the use of SOM as a clustering method, with each neuron acting as a cluster. For example, a 5x5 neuron SOM would generate 25 clusters, which distinguishes itself from other methods by having topological relationships *among* clusters explicitly represented. One further has to keep in mind that the SOM method never involves computation of direct document-document similarities. Instead, document vectors are treated as samples from the continuous n-dimensional input space (i.e., terms have continuous weights, not discrete counts) and the result of SOM training is a *model* through which document vectors can be mapped by finding the most similar neuron vector and thereby determining a 2D location for each document. Therefore, the SOM model could be used to map the original document vectors, but its true power (and distinction from the other methods used in the study) lies in the ability to map *any* document, whether or not it was contained in the original training data set. That is computationally unproblematic and millions of documents could quickly be mapped out in this manner.

Meanwhile, the initial generation of the underlying detailed model of the document space is computationally expensive. SOM use in this study aimed for a balance between the amount of geometric/topological distinctions (i.e., number of neurons) and the semantic depth (i.e., number of dimensions). Initial experiments with SOM PAK (a standard implementation) indicated that use of the full set of 23,347 dimensions from the MeSH-by-document dataset was computationally unfeasible. Thus, we reduced the dimensionality of the input data by keeping the 2,300 most frequent MeSH terms, which allowed us to construct a SOM of 75,625 neurons (275x275). The resulting model can itself be the basis of visualization, without involving the document vectors as such (Figure 2).


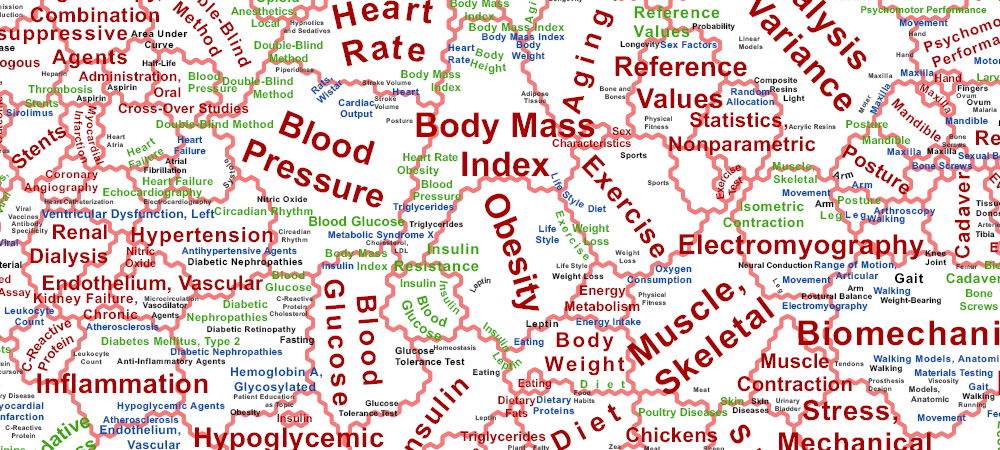


**Figure 2. Portion of a visualization of the biomedical knowledge domain derived from MeSH-based data through the SOM method.**

In order to allow some comparison to the other methods, the full set of MeSH-based document vectors was then mapped on the SOM by assigning each document to the best-matching neuron. Since the number of neurons was roughly double the number of clusters in the other solutions, adjacent neurons containing few documents were combined into clusters until each such cluster contained at least 25 documents. Together with those neurons already containing 25 documents, this resulted in 29,941 clusters partitioning the document set.

*Topic modeling (Topics):* The topic model – a recently-developed Bayesian model for text document collections – is considered a state-of-the-art algorithm for extracting semantic structure from text collections. The topic model automatically learns a set of thematic topics (in the form of lists of words) that describe a collection, and assigns a small number of these topics to each and every document in the collection. The topic model evolved from earlier dimensionality reduction techniques such as LSA, and could be considered as a probabilistic version of LSA .

Unlike the co-word and LSA calculations, topic modeling was only run on the term-document data, and not on the MeSH-document data. Some additional preprocessing was done on these data before they were subjected to the topic modeling algorithms. First, 131 topically uninteresting but frequently occurring words were removed from the data (e.g., words such as 'study', 'studies', 'result', 'results', etc.) Next, all terms that occurred fewer than 50 times across the entire corpus were removed. This reduced word-document set retained all 2,153,769 documents, but reduced the number of unique tokens to 65,776. The total number of word-document triples was 243,724,698 (88% of the original number), thus giving an average length of 113 words per document.

A standard Gibbs-sampled topic model was run on this reduced term-document collection. Three separate topics models were learned at the following topic resolutions: T=500, T=1000 and T=2000 topics. These topics models were run for: 1600, 1500 and 1200 iterations (i.e. entire sweeps through the corpus), respectively. Dirichlet prior hyperparameter settings of β=0.01 and α=0.05*N/(D*T) were used.[[3]](#footnote-4)

From the results of these three models, the top 20 most similar documents for each of the 2,153,769 documents in the corpus were computed. A topic-based similarity metric was used, using an equal weighting of the T=500, T=1000 and T=2000 topic models. Specifically, the similarity between documents A and B were calculated as:

sim*(A,B) = 1 - ( L_1_(A_500_-B_500_) + L_1_(A_1000_-B_1000_) + L_1_(A_2000_-B_2000_) )/6*

where *L_1_* is the L_1_ norm (the sum of the absolute values of the vector entries), and *A_500_*, etc. is the distribution over T=500, etc. topics of document A. We spot-checked a small number of similarities using PubMed, and this spot-checking indicated good agreement and consistency with PubMed.

*PubMed related articles (pmra):* When one does a search on PubMed/MEDLINE and displays the results in Summary mode, most records show a Related Articles link.

“The Related Articles link is as straightforward as it sounds. PubMed uses a powerful word-weighted algorithm to compare words from the title and abstract of each citation, as well as the MeSH headings assigned. The best matches for each citation are pre-calculated and stored as a set.” [[4]](#footnote-5)

The *pmra* algorithm used to pre-calculate these Related Articles has been through sufficient testing and review to have been accepted for use in PubMed, and is thus a *de facto* standard. It seemed wise to add a set of calculations based on this method to the project as an additional point of comparison.[[5]](#footnote-6)

Dave Newman (UCI) wrote and ran a script that queried PubMed for these pre-calculated matches for each document in our corpus. This script returned a rank-ordered list of the Related Articles, which was then post-processed to limit the lists to only documents that were in the corpus. After post-processing, these lists contained from 2 to 20 Related Articles for each article in the corpus, listed in rank order, but without the actual *pmra* similarity values.

In order to compare a map of science using these Related Articles to the other maps, it was necessary to convert the rank-ordered lists of relationships into similarity values. We estimated the Related Articles similarity values as

*PMRA_A,B_ = 0.02*(51 – rank_A,B_)*

for all articles *B* related by *rank_A,B_* to article *A*. Thus, for any article *i*, the first ranked Related Article was assigned a similarity value of 1.00, the second a similarity value of 0.98, etc. We cannot note strongly enough that these are not the original similarity values calculated using the *pmra* method, but are rather estimated values from rank orders. This approach is thus inherently different in a fundamental way from any of the other approaches tested.

***Additional filtering of text-based similarities***

The similarity approaches detailed above each generated similarity listings with different numbers of similarity pairs. For the tfidf and LSA approaches the top 50 similarities per article were retained, while only the top 20 were retained for the topic modeling and pmra approaches, and the top 15 retained for the bm25 measure. We applied an additional filtering step to each of these similarity listings. Large similarity files (over 25 million similarity values) are too large for our clustering routine to handle efficiently. Regarding the filtering of similarities, although many would argue that the effect of filtering is to lose information and thus reduce accuracy, our experience has been that filtering of similarity lists acts as a noise reduction technique, and actually increases the accuracy of a cluster solution .

Our method for this filter is to generate a top-n similarity file from the larger similarity listings generated by the steps above. The premise behind this is that papers that are more strongly linked (i.e., those with higher similarities) should contribute more edges to the solution space. Papers with small similarities should not contribute as many edges because they are not very similar to anything. For example, within a given corpus, consider paper A whose average similarity value over its top15 similarities is 0.85, and paper B whose average similarity over its top15 similarities is 0.15. Note that we picked paper A because it has the highest average top15 similarity value, and paper B because it has the lowest average top15 similarity value within the set. These two papers thus define the top15 similarity range; paper A contributes 15 edges to the similarity file, and paper B contributes only 5 edges to the similarity file. We then scale all other papers between these two based on *log(avg(top15 sim))*. Each paper thus contributes between 5 and 15 edges to the similarity file. We then de-duplicated all (A:B – B:A) pairs for efficiency, and used these top-n similarity files as input to the clustering steps. This filtering step was applied to all of the similarity listings mentioned above with the exception of the SOM and the *pmra* approach.

For the pmra approach, given that the original distribution of similarities ranged from 2-20, rather than from the desired 5-15, we simply mapped one distribution to the other as:

*S_NEW_ = round(0.5*S_ORIG_ + 3.25)*

where *S_ORIG_* is the original number of similarities and *S_NEW_* is the new number of similarities. The final *pmra* top-n similarity file contained 18,511,515 document pairs, which is very similar to the final number of pairs obtained using the other approaches.

***Clustering***

Similarity files from each of the similarity approaches above were run through a standardized and very robust clustering process to generate sets of document clusters. The same clustering method was used for all similarity approaches; thus the clustering method should not contribute to any variability in the final results. The clustering process was comprised of four main steps:

1. The DrL[[6]](#footnote-7) (formerly VxOrd) graph layout routine was run using a similarity file as input, and using a cutting parameter of 0.975 (maximum cutting). DrL uses a random walk routine and prunes edges based on degree and edge distance; long edges between nodes of high degree are preferentially cut. A typical DrL run using an input file of 2M articles and 15M edges will cut approximately 60% of the input edges, where an edge represents a single document-document similarity pair from the original similarity file. At the end of the layout calculation, each article has an x,y position, and roughly 40% of the original edges remain.
2. Papers were assigned to clusters using an average-linkage clustering algorithm . The average-linkage clustering algorithm uses the article positions (x,y) and remaining edges to assign papers to clusters. Once the clusters are generated, the full list of pairs of papers that co-occur in a cluster are generated for each solution. For example, if papers A, B, C, and D are in a cluster together, the set of pairs will be AB, AC, AD, BC, BC, and CD.
3. Steps (1-2) were run 10 separate times using 10 different random starting seeds for DrL, and thus giving rise to 10 unique cluster solutions for the same similarity file. Different starting seeds (i.e., different starting points for the random walk) will give rise to different graph layouts and different (but typically highly overlapping) sets of remaining edges. We use these differences to our advantage in this clustering process.
4. Those paper pairs that appear in 4 or more out of the 10 DrL solutions are considered to be the robust pairs, and are listed in a separate file.[[7]](#footnote-8) This list of pairs is then used as the input edges to the same average-linkage clustering algorithm used in step (2). The numbers of solutions in which each paper pair appeared were input to the average-linkage algorithm along with the paper pairs; scores of 6/10 or less could be used to start clusters or to add single papers to existing clusters, but a score of 7/10 or higher was required for the paper pair to merge clusters. Each cluster emerging from this process was referred to as a level 0 cluster.
5. Logic dictates that a cluster should have a minimum size; otherwise there is not enough content to differentiate it from other clusters. In our experience, a cluster should contain a minimum of approximately five papers per year (or 25 papers over the five year length of the corpus) to be considered topical.[[8]](#footnote-9) Thus we take all clusters with fewer than 25 papers, and aggregate them. This is done by calculating K50 similarities between all pairs of level 0 clusters, and then aggregating each small cluster (< 25 papers) with the cluster to which it has the largest K50 similarity until no clusters with < 25 papers remain. K50 values are calculated from aggregated modified frequency values (the *1/log(p(C+1))* values) where available, and from the aggregated top-n similarity values in all other cases. The resulting aggregated clusters are known as level 1 clusters.

This clustering process is very robust, and is designed to only cluster paper pairs with a high signal-to-noise ratio. For example, using a citation-based model of roughly 2.1M documents and 16M edges, the adjusted Rand index between pairs of single DrL solutions was 0.32, while the adjusted Rand index between pairs of 10xDrL solutions was over 0.80. This huge increase in the adjusted Rand index justifies the use of the more complex 10xDrL solution, and exemplifies its robustness.

**Accuracy Metrics**

Given that the main purpose of this study was to determine which similarity approach would create the most accurate map of the medical sciences, we needed a way to measure the relative accuracies of each cluster solution. We settled on two measures – the first measures the within-cluster textual coherence, and the second is a new measure designed to show which similarity approach best concentrates grant-to-article linkages. We note that the notion of a research front includes both topical and social components, and further note that both of these measures focus on the topical content rather than the social aspects of the document clusters. We are not aware of an accuracy measure based on the social component of document clusters; such a measure would be a welcome addition to the literature.

***Textual coherence***

The quantity that is used here to measure textual coherence is the Jensen-Shannon (JSD) divergence . It is used to quantify the distance (or divergence) between two (or more) probability distributions. JSD[[9]](#footnote-10) is calculated for each document from the word probability vector for that document, and from the word probability vector for the cluster in which the document resides as:

*JSD(p, q) = ½ D_KL_(p, m) + ½ D_KL_(q, m)*

where *m = (p+q)/2* and *D_KL_(p, m) = ∑ (p_i_ log (p_i_/m_i_))*

and *p* is the frequency of a word in a document, *q* is the frequency of the same word in the cluster of documents, and *D* is the well-known Kullback-Leibler divergence. JSD is calculated for each cluster as the average JSD value over all documents in the cluster.

JSD is a divergence measure, meaning that if the documents in a cluster are very different from each other, using different sets of words, the JSD value will be very high, or close to 1.0. Clusters of documents with similar sets of words – a less diverse set of words – will have a lower divergence. The use of JSD is not limited to sets of words, but is commonly used in mathematical statistics and statistical physics applications , and more recently in bioinformatics .

JSD varies with cluster size. For example, a cluster with 10 very different documents will have a larger set of unique elements, and thus a higher divergence value than a cluster with only 3 very different documents. The maximum possible JSD values for various cluster sizes will occur when the documents in the cluster have completely different sets of elements. These maximum divergence clusters can be approximated, for a particular corpus, by forming random clusters of documents from that corpus. We have calculated JSD values for randomly formed clusters of different sizes from the study corpus . Each measured divergence value is an average of the divergence values from a very large number of random clusters (e.g., 5000 random clusters of size 20, 5000 random clusters of size 100, 1000 random clusters of size 500). A curve fit of the measured values was used to estimate the JSD values for every cluster size from 2 to 1000.

Coherence is calculated from divergence values for each cluster *i* as:

*Coh_i_ = JSD(rand) _i_ – JSD(actual) _i_*

where *JSD(rand)* is the random divergence for the particular cluster size. The average coherence value for an entire cluster solution is then calculated as a weighted average:

*Coh = ∑n_i_*Coh_i_ / ∑n_i_ .*

summed over all clusters *i* where *n_i_* is the size of cluster *i*.

Other studies that have measured within-cluster textual coherence include Braam et al. and Jarneving , although both used different mathematical formulations of coherence.

***Concentration of grant-to-article linkages***

One of the challenges of comparing text-based and citation-based cluster solutions of a particular corpus is to find a metric that is independent of both text and citation, and that can be considered unbiased. Although only citation-based work is reported in this article, our complete study included both types of approaches. Given that a textual coherence is likely to favor text-based solutions over citation-based solutions, we needed a second accuracy measure, and one that was less biased toward either text or citation. During informal conversations one of the authors suggested that the grant acknowledgements mined from MEDLINE might be a suitable dataset from which to design such a metric. A grant-to-article linkage dataset from a previous study , consisting of a matched set of grant numbers and PMID, was available for such a purpose.

In order to measure concentration, one must limit the basis set to those elements that can actually show a concentrated solution. For example, grants that have only produced one article cannot differentiate between cluster solutions. Thus, we limited the grant-to-article linkage set to those grants that have produced a minimum of four articles. The resulting basis set thus consisted of 571,405 separate links between 262,959 unique articles and 43,442 NIH grants.

The premise for using these grant-to-article linkages as a metric for measuring the accuracy of a cluster solution is the assumption that the papers acknowledging a single grant should be highly related, and should be concentrated in a cluster solution of the document space. Using this approach, a cluster solution giving a higher concentration of grants would be considered to be more accurate than one with a lower concentration value. In addition, since grants are not inherently tied to the clustering of scientific articles either by text or by citations, we consider a grant-based metric to be unbiased.

We considered several different measures based on grant-to-article linkages including a standard Herfindahl (or concentration) index and precision-recall curves. The Herfindahl index had the advantage that it could be calculated on a grant-by-grant basis and then averaged over grants, thus ensuring high specificity. Its disadvantage is that it would give a single number for comparison. By contrast, a precision-recall method gives curves that show a distribution of metric values. The disadvantage of this approach is loss of grant-to-grant specificity; articles in a cluster might refer to different grants rather than the same grant.

We settled on the precision-recall measure so that we could see the distribution of the results. In this formulation of precision-recall one orders all clusters in a solution by the fraction of the articles in the cluster that reference a particular set of grants, and then generates a traditional precision-recall curve. In this case, recall would be the cumulative fraction of the links present in the ordered clusters (SumLink/TotLink in Table 3), and precision would be the cumulative fraction of the articles in the set of clusters retrieved that referenced the set of grants (SumArtL/SumArt in Table 3). In this setting, precision can be equated with concentration. This measure should be particularly well suited to comparing maps whose stated purpose is portfolio analysis since the portfolios that will be analyzed are most often tied to funded grants.

**Table 3. Example of cumulative precision-recall calculation based on grant-to-article linkages. Assume that the total number of linkages (TotLink)** available is 2000.

| Clust | Art | ArtL | Links | Frac | SumArt | SumArtL | SumLink | R | Pr |
| --- | --- | --- | --- | --- | --- | --- | --- | --- | --- |
| 1 | 100 | 90 | 150 | 0.90 | 100 | 90 | 150 | 0.075 | 0.900 |
| 2 | 100 | 80 | 130 | 0.80 | 200 | 170 | 280 | 0.140 | 0.850 |
| 3 | 100 | 70 | 120 | 0.70 | 300 | 240 | 400 | 0.200 | 0.800 |

Art – number of articles in cluster, ArtL – number of articles in clusters linked to grants, Links – number of unique links to the ArtL (articles can be linked by more than one grant), Frac – ArtL/Art, Sum* – cumulative sums, R – recall = SumLink/TotLink, Pr – precision = SumArtL/SumArt.

**References**

Blei, D. M., Ng, A. Y., & Jordan, M. I. (2003). Latent Dirichlet Allocation. *Journal of Machine Learning, 3*, 993-1022.

Boyack, K. W. (2009). Linking grants to articles: Characterization of NIH grant information indexed in Medline. *12th International Conference of the International Society for Scientometrics and Informetrics*, 730-741.

Boyack, K. W., & Klavans, R. (2010). Co-citation analysis, bibliographic coupling, and direct citation: Which citation approach represents the research front most accurately? *Journal of the American Society for Information Science and Technology, 61*(12), 2389-2404.

Boyack, K. W., Klavans, R., & Börner, K. (2005). Mapping the backbone of science. *Scientometrics, 64*(3), 351-374.

Braam, R. R., Moed, H. F., & Van Raan, A. F. J. (1991). Mapping of science by combined co-citation and word analysis I. Structural aspects. *Journal of the American Society for Information Science, 42*(4), 233-251.

Gorrell, G., & Webb, B. (2005). Generalized Hebbian algorithm for incremental latent semantic analysis. *9th European Conference on Speech Communication and Technology*, 1325-1328.

Grosse, I., Bernaola-Galvan, P., Carpena, P., Roman-Roldan, R., Oliver, J., & Stanley, H. E. (2002). Analysis of symbolic sequences using the Jensen-Shannon divergence. *Physical Review E, 65*, 041905.

Jarneving, B. (2007). Bibliographic coupling and its application to research-front and other core documents. *Journal of Informetrics, 1*(4), 287-307.

Klavans, R., & Boyack, K. W. (2006a). Identifying a better measure of relatedness for mapping science. *Journal of the American Society for Information Science and Technology, 57*(2), 251-263.

Klavans, R., & Boyack, K. W. (2006b). Quantitative evaluation of large maps of science. *Scientometrics, 68*(3), 475-499.

Kohonen, T. (1995). *Self-Organizing Maps*: Springer.

Kohonen, T., Hynninen, J., Kangas, J., & Laaksonen, J. (1996). *SOM PAK: The Self-Organizing Map program package. Technical Report A31*: Helsinki University of Technology, Laboratory of Computer and Information Science.

Landauer, T. K., & Dumais, S. T. (1997). A solution to Plato's Problem: The Latent Semantic Analysis theory of acquisition, induction, and representation of knowledge. *Psychological Review, 104*(2), 211-240.

Lin, J. (1991). Divergence measures based on Shannon entropy. *IEEE Transactions on Information Theory, 37*(1), 145-151.

Lin, J., & Wilbur, W. J. (2007). PubMed related articles: A probabilistic topic-based model for content similarity. *BMC Bioinformatics, 8*, 423.

Martin, S., Brown, W. M., Klavans, R., & Boyack, K. W. (2011). OpenOrd: An open-source toolbox for large graph layout, *Conference on Visualization and Data Analysis 2011*. San Francisco, CA.

Newman, D. J., & Block, S. (2006). Probabilistic topic decomposition of an eighteenth-century American newspaper. *Journal of the American Society for Information Science and Technology, 57*(6), 753-767.

Sims, G. E., Jun, S.-R., Wu, G. A., & Kim, S.-H. (2009). Alignment-free genome comparison with feature frequency profiles (FFP) and optimal resolutions. *Proceedings of the National Academy of Sciences of the USA, 106*(8), 2677-2682.

Sparck Jones, K., Walker, S., & Robertson, S. E. (2000a). A probabilistic model of information retrieval: Development and comparative experiments. Part 1. *Information Processing & Management, 36*(6), 779-808.

Sparck Jones, K., Walker, S., & Robertson, S. E. (2000b). A probabilistic model of information retrieval: Development and comparative experiments. Part 2. *Information Processing & Management, 36*(6), 809-840.

1. available at <http://www.dcs.shef.ac.uk/~genevieve> [↑](#footnote-ref-2)
2. One modification to the cosine code was made for the LSA calculation. Since the LSA output is much denser than the co-word data, but with many fewer dimensions, all entries (zero and non-zero) for each document were stored in fixed-width arrays, and the dot products of the arrays were calculated using basic Linear Algebra Subprograms. [↑](#footnote-ref-3)
3. Examples of topics can be found at <http://www.ics.uci.edu/~newman/katy/> [↑](#footnote-ref-4)
4. <http://www.nlm.nih.gov/bsd/disted/pubmedtutorial/020_190.html> [↑](#footnote-ref-5)
5. [http://www.ncbi.nlm.nih.gov/bookshelf/br.fcgi?book=helppubmed&part=pubmedhelp#pubmedhelp.Computation_of_Relat](http://www.ncbi.nlm.nih.gov/bookshelf/br.fcgi?book=helppubmed&part=pubmedhelp#pubmedhelp.Computation_of_Relat) [↑](#footnote-ref-6)
6. Sandia National Laboratories has recently renamed DrL to OpenOrd, which is freely available at <http://www.cs.sandia.gov/~smartin/software.html>. [↑](#footnote-ref-7)
7. The original criteria we used for the final clustering was that a paper pair appear in 6/10 DrL solutions to be retained for the final clustering. This criterion works very well for citation-based approaches . However, for the text-based approaches we found that this criterion was too stringent, and resulted in coverages that were, on average, 10% less than those for citation-based approaches. We thus experimented with the criteria, and found that a criterion of 4/10 to list the pair, combined with a criterion of 7/10 to merge clusters, gave coverages that were roughly equivalent to those obtained from the citation-based approaches, while maintaining a similar cluster size distribution. [↑](#footnote-ref-8)
8. This is based on an old, undocumented assumption among many bibliometricians that there must be a critical mass of around 5 papers per year in a cluster for the cluster to represent a specific and measurable topic in science. We have chosen to aggregate small clusters to this level, but others may choose to handle small clusters in a different manner. There is no consensus in the community as to how the issue of small clusters should be dealt with. [↑](#footnote-ref-9)
9. We use the simplified JSD formulation for two distributions of equal weights used in Sims et al. (2009), also found on Wikipedia (<http://en.wikipedia.org/wiki/Jensen-Shannon_divergence>). [↑](#footnote-ref-10)
